# Long-Term Outcomes of Surgical Resection of Pathologically Confirmed Isolated Para-Aortic Lymph Node Metastases in Colorectal Cancer: A Systematic Review

**DOI:** 10.3390/cancers14030661

**Published:** 2022-01-28

**Authors:** Maurizio Zizzo, Maria Pia Federica Dorma, Magda Zanelli, Francesca Sanguedolce, Maria Chiara Bassi, Andrea Palicelli, Stefano Ascani, Alessandro Giunta

**Affiliations:** 1 Surgical Oncology Unit, Azienda Unità Sanitaria Locale-IRCCS di Reggio Emilia, 42123 Reggio Emilia, Italy; MariaPiaFederica.Dorma@ausl.re.it (M.P.F.D.); Alessandro.Giunta@ausl.re.it (A.G.); 2 Pathology Unit, Azienda Unità Sanitaria Locale-IRCCS di Reggio Emilia, 42123 Reggio Emilia, Italy; Magda.Zanelli@ausl.re.it (M.Z.); Andrea.Palicelli@ausl.re.it (A.P.); 3 Pathology Unit, Azienda Ospedaliero-Universitaria, Ospedali Riuniti di Foggia, 71122 Foggia, Italy; francesca.sanguedolce@unifg.it; 4 Medical Library, Azienda Unità Sanitaria Locale-IRCCS di Reggio Emilia, 42123 Reggio Emilia, Italy; MariaChiara.Bassi@ausl.re.it; 5 Hematology Unit, CREO, Azienda Ospedaliera di Perugia, University of Perugia, 06129 Perugia, Italy; s.ascani@aospterni.it; 6 Pathology Unit, Azienda Ospedaliera S. Maria di Terni, University of Perugia, 05100 Terni, Italy

**Keywords:** colorectal cancer, para-aortic, lymph nodes, metastases, surgery, outcome

## Abstract

**Simple Summary:**

Para-aortic lymph node (PALN) metastases represent patterns of initial recurrence in only 2–6% CRC patients. Lack of consensus has impaired an unambiguous statement for PALN recurrence resection. Our systematic review identified 59.4–68% 3-year OS rate and 53.4–87.5% 5-year OS rate, with a 25–84 months median OS, 26.3–61% 3-year DFS rate and 0–60.5% 5-year DFS rate, with a 14–24 month median DFS, in patient undergoing isolated PALNM resection. Overall, 62.1% re-recurrence rate ranged from 43.8% to 100%. Although PALNMs resection in CRC patients may be considered a feasible and beneficial option, no conclusions or recommendations can be provided, taking into account the current evidence. Further randomized, possibly multicenter trials are strongly recommended and mandatory in order to confirm our results and clearly identify patient selection criteria.

**Abstract:**

Background: Para-aortic lymph node (PALN) metastases represent patterns of initial recurrence in only 2–6% CRC patients, after an estimated 23–28 month time interval. An increasing trend towards curative surgery has been witnessed in patients presenting with controlled PALN recurrence. Nevertheless, lack of consensus has impaired an unambiguous statement for PALN recurrence resection. Methods: We performed a systematic literature review following the Preferred Reporting Items for Systematic Reviews and Meta-Analyzes (PRISMA) guidelines, which led us to gain deeper insight into the prognostic factors and long-term outcomes after resection for synchronous or metachronous pathologically confirmed CRC isolated para-aortic lymph node metastases (PALNM). Pubmed/MEDLINE, Embase, Scopus, Cochrane Library and Web of Science databases were used to search all related literature. Results: The nine articles included covered a study period of 30 years (1988–2018), with a total of 161 patients. At presentation, most primary CRCs were located in the colon (74%) and 95.6%, 87.1% and 76.9% patients had T3–T4, N1–N2 and well/moderately differentiated CRC, respectively. We identified a 59.4–68% 3-year OS rate and 53.4–87.5% 5-year OS rate, with a 25–84 months median OS, 26.3–61% 3-year DFS rate and 0–60.5% 5-year DFS rate, with a 14–24 month median DFS. Overall, 62.1% re-recurrence rate ranged from 43.8% to 100%. Conclusions: Although PALNMs resection in CRC patients may be considered a feasible and beneficial option, no conclusions or recommendations can be made taking into account the current evidence. Therefore, further randomized, possibly multicenter trials are strongly recommended and mandatory if we want to have our results confirmed and patient selection criteria clearly identified.

## 1. Introduction

Colorectal cancer (CRC) is the third most commonly occurring cancer, which has become the second leading cause of cancer-related deaths worldwide [1,2]. In CRC patients, metastatic dissemination represents the most frequent cause of death, as approximately 25% patients show CRC metastases at diagnosis [1].

As approximately 50% patients develop hepatic metastases in the course of the disease, the liver represents the most common site of CRC metastases [1]. About 20% of patients affected by hepatic metastases show a potentially resectable disease [1]. Following resective surgery of isolated hepatic metastases, low survival rates were reported (25–58% at 5 years, 17–28% at 10 years) [1], and the lung ranks second (10–15% patients with CRC at diagnosis) [1]. After the resection of isolated pulmonary metastases, survival rates range between 32% and 68% at 5 years and between 11% and 34% at 10 years [1].

Having been previously defined as retroperitoneal lymph nodes (RLN) recurrence, para-aortic lymph node (PALN) metastases represent patterns of initial recurrence in only 2–6% CRC patients, after an estimated 23–28 month time interval [3].

Although CRC hepatic and pulmonary metastases require standardized management, how to treat lymph node recurrence (either isolated or combined with other CRC metastatic lesions) remains an open question [1,2,3]. Some authors have made a distinction between retroperitoneal local recurrence and RLN recurrence [3]. Moreover, although complex protocols have been suggested, none of them have been fully validated and adopted [3]. An increasing trend towards curative surgery has been witnessed in patients presenting with controlled RLN recurrence [3]. Nevertheless, lack of consensus impaired an unambiguous statement for RLN recurrence resection [3].

A thorough systematic review of literature led us to achieve deeper knowledge of prognostic factors and long-term outcomes after resection for synchronous or metachronous pathologically confirmed CRC isolated para-aortic lymph node metastases (PALNM).

## 2. Materials and Methods

### 2.1. Search Strategy

We performed a systematic literature review in compliance with Preferred Reporting Items for Systematic Reviews and Meta-Analyzes (PRISMA) guidelines. According to the gold standard for literature search related to surgical reviews [4], the following databases were searched: PubMed/MEDLINE, Embase, Scopus, Cochrane Library (Cochrane Database of Systematic Reviews, Cochrane Central Register of Controlled Trials-CENTRAL) and Web of Science (Science and Social Science Citation Index). Combination of non-MeSH/MeSH terms was as follows:

- PubMed/MEDLINE

(Para-aortic OR Paraaortic) AND ((((“Colorectal Neoplasms” [Mesh])) OR ((Colorectal OR Colon OR Rectal) AND (cancer OR tumor OR tumour OR neoplasm))) AND ((“Lymph Nodes” [Mesh]) OR “Lymph Node Excision” [Mesh] OR Lymph node* OR Lymphadenectom* OR Lymph nodes dissection*)) Filters: English, Italian.

- Embase

(‘colon tumor’/exp OR (Colorectal OR Colon OR Rectal) AND (cancer OR tumor OR tumour OR neoplasm)) AND (‘lymph node dissection’/exp OR Lymph node* OR Lymphadenectom* OR Lymph nodes dissection*) AND (Para-aortic OR Paraaortic).

- Scopus

(TITLE-ABS-KEY (para-aortic OR paraaortic) AND TITLE-ABS-KEY ((colorectal OR colon OR rectal) AND (cancer OR tumor OR tumour OR neoplasm)) AND TITLE-ABS-KEY (lymph AND node* OR lymphadenectom* OR lymph AND nodes AND dissection*)) AND (LIMIT-TO (LANGUAGE, “English”) OR LIMIT-TO (LANGUAGE, “Italian”)).

- Cochrane Library

Para-aortic OR Paraaortic in Title Abstract Keyword AND (Colorectal OR Colon OR Rectal) AND (cancer OR tumor OR tumour OR neoplasm) in Title Abstract Keyword AND Lymph node* OR Lymphadenectom* OR Lymph nodes dissection* in Title Abstract Keyword—(Word variations have been searched).

- Web of Science

TOPIC: (Para-aortic OR Paraaortic) AND TOPIC: ((Colorectal OR Colon OR Rectal) AND (cancer OR tumor OR tumour OR neoplasm)) AND TOPIC: (Lymph node* OR Lymphadenectom* OR Lymph nodes dissection*).

Refined by: LANGUAGES: (ENGLISH) NO ITALIAN.

Final analysis was carried out in February 2021.

### 2.2. Terminology

To reach a better understanding of results, we adopted the following descriptions: RLN recurrence: histologically confirmed retroperitoneal lymph node metastasis/es, without local CRC recurrence, in an area which is laterally limited by the ureters, superiorly limited by celiac area and inferiorly limited by iliac vessels [5].

PALNM: histologically confirmed recurrence in lymph nodes surrounding abdominal aorta and inferior vena cava, with lateral extension to edge of the psoas major muscles, superior extension to the crura of the diaphragm and caudal extension to mid common iliac vessels [6].

RLN recurrence and PALNM were adopted by Authors to define the same pathological condition.

PALNM type A: A1—Lymph node metastases located in the area of the aortic hiatus, about 4–5 cm in width surrounded by the medial crus of diaphragm (these nodes are located within median arcuate ligament of diaphragm); A2—Lymph nodes metastases located in the area from the uppermost part of the origin of the celiac trunk to the lower margin of the left renal vein [6].

PALNM type B: B1—Lymph node metastases located in the area from the lower margin of the left renal vein to the uppermost part of the origin of the inferior mesenteric artery; B2—Lymph nodes metastases located in the area from the upper margin of the origin of the inferior mesenteric artery to the aortic bifurcation [6].

Isolated PALNM: Lymph node metastases when there are no CRC metastatic sites other than PALNs [7].

Combined CRC metastases: Lymph node metastases when one or more sites have CRC metastases other than PALNs [8].

Synchronous PALNM: PALNM identified and resected concurrently with primary CRC [8].

Metachronous PALNM: PALNM identified and resected after resection of primary CRC [8].

PALN dissection (PALND): separation and removal of lymphatic tissue from the para-aortic vessels.

PALNM resection: excision of PALNM through PALND.

### 2.3. Inclusion Criteria

Our search was limited to articles published from January 2000 to February 2021. The selection included just scientific papers in English or Italian language.

Our systematic review covered population studies (case series, case–control studies, cohort studies, controlled clinical trials and randomized clinical trials) which included 5 or more adult patients (over 18 years of age), who underwent surgical resection for pathologically confirmed CRC isolated PALNM. We ruled out abstracts, posters, case reports, previously published systematic reviews and/or meta-analyses and population studies analysing less than five patients.

Our review ruled out articles that examined mixed clinical populations, isolated PALNM plus combined CRC metastases, or exclusively combined CRC metastases populations, as well as mixed pathologically populations (pathologically positive plus pathologically negative PALNM) after PALND.

In addition, articles that reported or led to data on survival (overall survival-OS-rate and/or median survival) and disease-free survival (disease-free survival-DFS-rate and/or median disease-free survival) were included, and we ruled out articles that did not lead to data related to survival and disease-free survival. Moreover, reference lists of included studies were manually scanned to further identify any potentially relevant articles.

### 2.4. Data Extraction

The papers were selected and identified by two independent reviewers (M.Zi. and M.C.B.) based on title, abstracts, keywords and full-texts. The following information was collected from the papers used in this study: demographic data [author’s surname and year of publication, study period, study type, study Center, population size, gender and age, primary CRC location, CEA and CA 19.9 levels at PALNM resection, chemotherapy (CT) and radiotherapy]; histopathological data [primary CRC T and N stage, primary CRC histology]; PALNM data [timing of presentation, anatomical location, PALN harvested, PALNM retrieved]; outcomes data [mortality rates, median overall survival, disease-free survival rates, median disease-free survival, follow-up duration, recurrence]. All collected results were eventually reviewed by a third independent reviewer (M.P.F.D.).

### 2.5. Quality Assessment

The Newcastle-Ottawa quality assessment scale (NOS) was used to assess the quality of each study [9]. The thresholds for converting the Newcastle-Ottawa scales to AHRQ standards are as follows (good, fair, and poor): (i) good quality: 3 or 4 stars in selection domain AND 1 or 2 stars in comparability domain AND 2 or 3 stars in outcome/exposure domain, (ii) fair quality: 2 stars in selection domain AND 1 or 2 stars in comparability domain AND 2 or 3 stars in outcome/exposure domain; (iii) poor quality: 0 or 1 star in selection domain OR 0 stars in comparability domain OR 0 or 1 stars in outcome/exposure domain.

## 3. Results

### 3.1. Search Results and Study Characteristics

**Figure 1:**
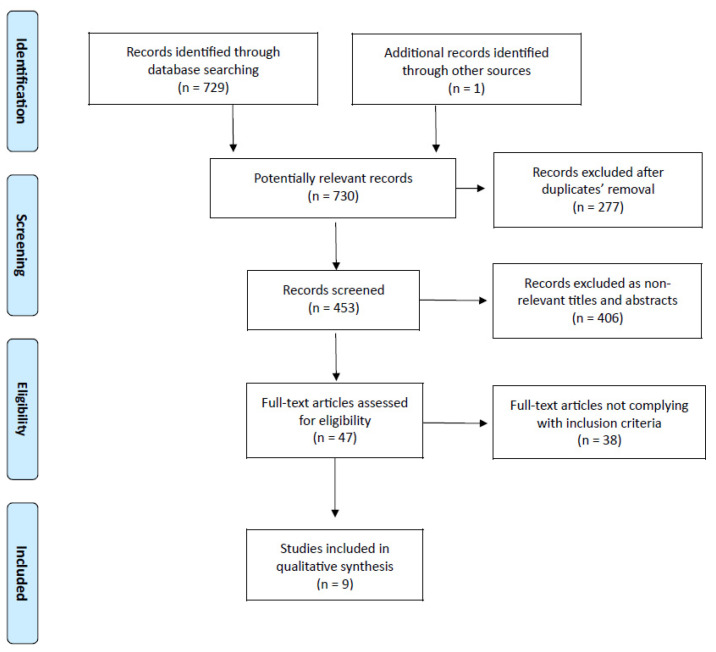
PRISMA flow chart of literature search.

A final literature search conducted in February 2021 helped to identify 729 studies of potential interest [PubMed/MEDLINE: 217 records; Embase: 120 records; Scopus: 170 records; Cochrane Library: 12 records; Web of Science: 210 records] (Figure 1). One additional record was found using another source (References list).

After ruling out 277 duplicate publications, a further analysis involved 453 studies, out of which 406 were considered to not be relevant for titles and abstracts, while 47 full-text articles were assessed for eligibility. After removing 38 studies that did not comply with the inclusion criteria, nine studies (all retrospective, most of them being of good quality, see Supplementary material-Table S1) underwent qualitative synthesis [5,10,11,12,13,14,15,16,17].

### 3.2. General Population Characteristics

Table 1 shows demographic features of the populations under analysis. The nine articles included covered the time period of 1988–2018, gathering a total of 161 patients [5,10,11,12,13,14,15,16,17]. Male prevalence (82/146 = 56.2%; 7 out of 9 studies) was recorded in addition to a 55.5–63 year median age [5,10,11,12,13,14,15,16,17]. At presentation, most primary CRCs were located in the colon (108/146 = 74%; 7 out of 9 studies) [5,10,11,12,13,14,15,16,17]. Almost all patients underwent adjuvant chemotherapy for PALNM (134/151 = 88.7%; 8 studies out of 9 ones) [5,10,11,12,13,14,15,16,17].

### 3.3. Primary CRC Histopathology

Table 2 shows the primary CRC histopathology data. Overall, 95.6% patients showed T3-T4 primary CRC (109/114; five studies out of nine, taking into account that Min et al. considered T2-T3 as a single group), while 87.1% patients had N1-N2 primary CRC (54/62; 4 studies out of 9 ones) [5,10,11,12,13,14,15,16,17].

Moreover, 76.9% patients presented with well/moderately differentiated CRC (100/130; 6 studies out of 9 ones) [5,10,11,12,13,14,15,16,17].

### 3.4. PALN Metastases

Table 3 shows the PALNM data. Almost all identified PALNM were synchronous with primary CRC (124/151 = 82.1%; 8 studies out of 9 ones) and type B (149/151 = 98.7%; eight studies out of nine) [5,10,11,12,13,14,15,16,17]. Average number of PALNM identified was 1–2 (five studies out of nine) out of a 3.5–11 average of the PALN harvested (4 studies out of 9 ones) [5,10,11,12,13,14,15,16,17].

**Table 1:** General population characteristics.

Author/Year	Study Period	Study Type	Patient Population, *n*	Gender, *n* (%)		Age, Median Years (Range)	Primary CRC Location, *n* (%)			CEA, Median ng/mL (Range)	CA 19.9, Median U/mL (Range)	Chemotherapy, *n* (%)			Radiotherapy, *n* (%)		
				Male	Female		Right Colon	Left Colon	Rectum			No	Neoadjuvant	Adjuvant	No	Neoadjuvant	Adjuvant
Min et al./2008	1992–2004	SC-RS	6	3 (50.0)	3 (50.0)	58.2 ^a^	3 (50.0)		3 (50.0)	22.04 ^a^	/	0	0	6 (100)	6 (100)	0	0
Choi et al./2010	1993–2006	SC-RS	24	13 (54.2)	11 (45.8)	52 (27–78) ^a^	11 (45.8)	4 (16.7)	9 (37.5)	/	/	1 (4.2)	0	23 (95.8)	24 (100)	0	0
Gagnière et al./2015	1997–2012	SC-RS	10	/	/	/	/	/	/	/	/	0	/	/	/	/	/
Song et al./2016	2004–2013	SC-RS	16	12 (75.0)	4 (25.0)	61 (45–79)	1 (6.2)	8 (50.0)	7 (43.8)	/	/	0	0	16 (100)	/	/	/
Ogura et al./2017	2004–2013	SC-RS	16	5 (31.3)	11 (68.7)	58.5 (39–82)	3 (18.8)	11 (68.8)	2 (12.4)	8.3 (2.6–154.2)	23.2 (6.4–313.3)	0	4 (25.0)	15 (93.8)	/	/	/
Bae et al./2018	1988–2009	SC-RS	49	29 (59.2)	20 (40.8)	57.5 ± 11.5 ^a^	6 (12.2)	43 (87.8)	0	23.7 ± 2.8 ^a^	/	/	/	47 (95.9)	/	/	/
Yamamoto et al./2019	2013–2018	SC-RS	5	/	/	/	/	/	/	/	/	0	0	5 (100)	/	/	/
Kim et al./2020	2004–2015	SC-RS	16	12 (75.0)	4 (25.0)	55.5 (42–73)	9 (56.3)		7 (43.7)	/	/	3 (18.7)	0	13 (81.3)	0	0	4 (25.0)
Sakamoto et al./2020	1986–2016	SC-RS	19	8 (42.1)	11 (57.9)	63 (46–74)	0	9 (47.4)	10 (52.6)	/	/	10 (52.6)	0	9 (47.4)	/	/	/

CRC = Colorectal cancer; CEA = Carcinoembryonic antigen; CA 19.9 = Carbohydrate antigen 19.9; SC-RS = Single-center retrospective study. ^a^ = mean.

**Table 2:** Primary CRC histopathology.

Author/Year	T, *n* (%)					N, *n* (%)			Histology, *n* (%)				
	T1	T2	T3	T4a	T4b	N0	N1	N2	Well Differentiated	Moderately Differentiated	Poorly Differentiated	Mucinous	Signet Ring Cell
Min et al./2008	0	6 (100)		0	0	0	6 (100)		6 (100)		0	0	0
Choi et al./2010	1 (2.1)	1 (2.1)	20 (83.3)	2 (8.3)		1 (2.1)	5 (20.9)	18 (75.0)	17 (70.8)		7 (29.2)		
Gagnière et al./2015	/	/	/	/	/	/	/	/	/	/	/	/	/
Song et al./2016	0	2 (12.5)	9 (56.3)	5 (31.2)		1 (6.2)	3 (18.8)	12 (75.0)	/	/	/	/	/
Ogura et al./2017	/	/	/	/	/	/	/	/	11 (68.8)		5 (31.2)		0
Bae et al./2018	0	1 (2.0)	43 (87.8)	5 (10.2)		/	/	/	4 (8.2)	34 (69.4)	6 (12.2)	5 (10.2)	0
Yamamoto et al./2019	/	/	/	/	/	/	/	/	/	/	/	/	/
Kim et al./2020	/	/	/	/	/	6 (37.5)	6 (37.5)	4 (25.0)	0	15 (93.8)	1 (6.2)	0	0
Sakamoto et al./2020	0	0	9 (47.4)	10 (52.6)		/	/	/	13 (68.4)		6 (31.6)		0

CRC = Colorectal cancer.

**Table 3:** PALN metastases.

Author/Year	Timing of Presentation, *n* (%)		Anatomical Location, *n* (%)		PALN Harvested, Median *n* (Range)	PALNM, Median *n* (Range)
	Synchronous	Metachronous	Type A	Type B		
Min et al./2008	0	6 (100)	2 (33.3)	4 (66.7)	/	/
Choi et al./2010	19 (79.2)	5 (20.8)	0	24 (100)	/	/
Gagnière et al./2015	/	/	/	/	/	/
Song et al./2016	16 (100)	0	0	16 (100)	3.5 (1–17)	1 (1–17)
Ogura et al./2017	16 (100)	0	0	16 (100)	/	1 (0–4)
Bae et al./2018	49 (100)	0	0	49 (100)	6.9 ± 5.2 ^a^	3.9 ± 4.0 ^a^
Yamamoto et al./2019	5 (100)	0	0	5 (100)	8 (1–23)	/
Kim et al./2020	0	16 (100)	0	16 (100)	/	1 (1–6)
Sakamoto et al./2020	19 (100)	0	0	19 (100)	11 (1–45)	2 (1–25)

PALN = Para-aortic lymph node. ^a^ = mean.

### 3.5. Long-Term Outcomes and Recurrences

Table 4 shows long-term outcomes and recurrence data. The 3-year OS rates were between 59.4% and 68% (4 studies out of 9 ones), while 5-year OS rates were between 53.4% and 87.5% (5 studies out of 9 ones) [5,10,11,12,13,14,15,16,17]. The median OS ranged between 25 and 84 months (7 studies out of 9 ones) [5,10,11,12,13,14,15,16,17]. On the contrary, 3-year DFS rates ranged between 26.3% and 61% (4 studies out of 9 ones) while 5-year DFS ranged between 0% and 60.5% (5 studies out of 9 ones) with a median DFS of 14–24 month (5 studies out of 9 ones) [5,10,11,12,13,14,15,16,17].

In terms of concerned patients with re-recurrence after surgery for isolated PALNM, the mean rate was between 43.8% and 100% (7 studies out of 9 ones), with a 62.1% mean re-recurrence pooled population (82/132; 7 studies out of 9 ones) [5,10,11,12,13,14,15,16,17]. With concern to re-recurrences, liver, lungs and PALN were found to be the primarily involved sites [5,10,11,12,13,14,15,16,17].

## 4. Discussion

Lymph nodes represent one of the most commonly affected sites of metastasis [18]. Tumours spread to lymph nodes via lymphatic vessels and this usually happens in order of proximity to the primary site [18]. Lymph node (N category) staging includes information on whether cancer has spread to regional lymph nodes and on the number of involved lymph nodes [18]. Regional lymph nodes, which are located along the course of major vessels that supply the large intestine, are designated based on the anatomical subsite of large bowel [18]. Lymph nodes, which are located outside the regional drainage area of a primary tumour, should be described as distant metastases (M category) [18].

With an incidence rate of less than 2%, PALN involvement in CRC is uncommon and American Joint Committee on Cancer (AJCC) considers it to be a disseminated stage IV disease, although Japanese Society for Cancer of the Colon and Rectum (JSCCR) consider regional PALNM to be a stage III disease [19,20].

Synchronous isolated PALNM was found to be 3.4% for right colon cancer, 3.9% for left colon cancer and 3.1% for rectal cancer [7]. Recording a 1.0–1.3% range, metachronous isolated PALNM is a rare condition, as PALNM is frequently followed by other distant site metastases [7]. In CRC, isolated PALNM has not been classified and it has been previously classified as retroperitoneal recurrence, a kind of locoregional recurrence [7]. Retroperitoneal recurrence does not only include PALNM, but also the growth of a tumor deposit or residual tumour from surgery [7]. Thus, isolated PALNM must be carefully investigated in terms of previous reports, in order to identify its management in the field of CRC [7]

As is the case for PALNM resection from other gastrointestinal tract cancers (e.g., gastric or pancreatic cancer), some authors have stressed that CRC PLANM resection can lead to better oncological outcomes in selected patients [21,22,23]. Indeed, some studies considered RLN metastases to be an extension of mesenteric lymph node metastases, or second-echelon regional lymph node metastases, with the potential for curative resection [5,10,11,12,13,14,15,16,17]. From this perspective, the involvement of major vessels in the retroperitoneum represents the only barrier for curative resection [16].

In our systematic review, we aimed to identify the long-term outcomes and prognostic factors related to surgical treatment of CRC isolated PALNM. In order to reduce potential confounding factors, we exclusively took into account manuscripts reporting surgical outcomes of isolated, pathologically confirmed CRC PALNM [5,10,11,12,13,14,15,16,17]. Therefore, we ruled out articles with mixed populations, isolated plus combined PALNM and pathologically positive plus pathologically negative PALNM. We identified 59.4–68% 3-year OS rate and 53.4–87.5% 5-year OS rate, with a 25–84 months median OS, 26.3–61% 3-year DFS rate and 0–60.5% 5-year DFS rate, with a 14–24 month median DFS.5,10–17 Overall, 62.1% re-recurrence rate ranged from 43.8% to 100% [5,10,11,12,13,14,15,16,17].

Three previously published systematic reviews had investigated the same topic we chose [7,8,24]. In 2011, Ho et al. studied a 110 patient population (isolated plus combined; surgery alone), identifying a 34–44 month median OS and a 17–21 month median DFS [24]. In 2016, Wong et al. investigated a patient population of 370 (isolated alone; surgical plus no surgical group); out of whom, 145 patients had synchronous metastases and 225 ones had metachronous metastases. Furthermore, 8 5-year OS and DFS seemed to be relatively similar; in the synchronous PALNM group, 5-year OS ranged from 22.7% to 65.7%, while DFS ranged from 17.6% to 40.2%; in the metachronous PALNM group, 5-year DFS ranged from 15% to 60%, while DFS ranged from 10% to 25.6% [8]. In 2018, Sasaki et al. analyzed an overall population of 227 patients (isolated plus combined; surgical plus no surgical group) [7]. The 3-year OS ranged from 60% to 100%, with a median 34–80 month OS for patients who underwent PALND [7]. However, findings from the aforementioned studies differ from our analysis, as they also took into account case reports or populations with less than five patients but, above all, also included patients who had undergone PALND based on radiological findings, although reported to be pathologically negative later.

The potential impact of a time lapse to presentation of metastases in PALNM patients represents a highly interesting topic. According to the literature, synchronous metastases in primary CRC tend to record a worse prognosis if compared to metachronous presentation [1]. Ruling out the study by Choi et al., which included a mixed synchronous/metachronous population, we recorded a 34–84 month median OS in a synchronous PALNM population and a 25–37 month median OS in metachronous PALNM [5,10,11,12,13,14,15,16,17]. On the other hand, DFS in both populations were recorded at similar rates (17–20.3 months in synchronous PALNM population versus 22–24 months in metachronous PALNM one) [5,10,11,12,13,14,15,16,17]. However, with regard to patient population heterogeneity, in addition to patient selection bias and different cancer biology (e.g., right colic cancer versus left colic cancer versus rectal cancer), the above mentioned results should be considered with caution.

In the selection of patients suitable for PALND, Albandar et al. emphasized the role of perioperative chemotherapy or chemoradiotherapy (CRT), as tumor regression or non-progression of other metastases after CT or CRT may mean a favourable biology, thereby identifying patients who are more likely to benefit from surgery, i.e., very few patients who undergo preoperative treatment and almost all those who have postoperative treatment [20]. However, none of the abovementioned studies provided an indication of pre- and post-operative CT/CRT or their consequences on subsequent surgical management [5,10,11,12,13,14,15,16,17]. Furthermore, only five of the nine authors reported the chemotherapy regimens they had used [10,11,12,13,14]. In particular, we identified eight different chemotherapy regimens in the absence of plain reasons for their indication (see Supplementary material-Table S2). Among the five abovementioned authors, only Ogura et al. analyzed impact of chosen chemotherapy regimen on survival: they identified an absence of statistical significance between no oxaliplatin/CPT-11 group and oxaliplatin/CPT-11 one (55.6% vs. 77.8% 5-year cancer-specific survival) [13]. Of the 9 studies, just Kim et al. reported patients (4) also undergoing adjuvant RT [16]. However, the authors did not describe both the RT schemes adopted and the possible influence of RT on survival [16].

Non-surgical management (CT and/or radiation therapy—RT) has been used as rescue therapy for PALNM patients.

In particular, only a few studies reported RT outcomes in PALNM patients [25,26,27,28]. Different RT techniques were described in four case series [25,26,27,28]. Kim et al. assessed the use of chemotherapy followed by stereotactic body RT using total SBRT doses that ranged from 36 to 51 Gy (median 48 Gy) in 7 metachronous PALNM patients [25]. They reported 1- and 3-year OS rates of 100% and 71.4% rates, respectively, with a 37 month median OS [25]. Yeo et al. administered 20 metachronous PALNM patients with three-dimensional conformal RT and 2 metachronous PALNM ones with helical tomotherapy, with concurrent CT [26]. The total dose was 63 Gy in 35 fractions (*n* = 12) or 55.8 Gy in 31 fractions (*n* = 8), with 1.8 Gy per fraction and 5 days/week [26]. 3- and 5-year OS rates recorded as 64.7% and 36.4% rates, respectively, while median OS was 41 months [26]. Lee et al. compared upfront RT versus RT following systemic therapy which used conventional 50–65 Gy RT or short-course 25 Gy RT in 52 metachronous PALNM patients [27]. Median OS was 41 months and the 2-year OS showed a 69.6% rate [27]. Isozaki et al. administered 34 metachronous PALNM patients with carbon-ion radiotherapy without concurrent CT [28]. A median 52.8 Gy total dose (48–52.8 Gy RBE) was administered [28]. According to Authors’ analysis, 2-, 3-, and 5-year OS rates were 83.3%, 63.0%, and 21.0%, respectively, with a 41.7 month median OS [28]. In a recent meta-analysis, the complete response rate ranged from 31% to 59.1%, while the partial response rate ranged from 17.6% to 57.1%, and the steady disease was recorded as 13.6–26.5% rates. Furthermore, seven 68.2–100% recurrence rates were reported and median DFS recorded 13–20 months [7].

In all of the included studies, the authors tried to identify prognostic factors in patient populations undergoing dissection for PALNM from CRC, with the aim of detecting prospective selection criteria (Table 5) [5,10,11,12,13,14,15,16,17]. According to the available data from the multivariate analyses, poor survival affects all patients who have the following: poorly differentiated/mucinous primary CRC, positive pN, >7 PALNM metastases and PALNM combined with metastases in other sites [5,10,11,12,13,14,15,16,17]. The same holds true for patients with metachronous PALNM and <24 month DFI [5,10,11,12,13,14,15,16,17]. On the contrary, a lower DFS was recorded for patients with >7 PALNM metastases, PALNM combined with metastases in other sites and a >5 ng/mL preoperative CEA [5,10,11,12,13,14,15,16,17].

Unfortunately, it was not possible to obtain details about the potential relevance of the resection margin on long-term outcomes. However, we believe that the resection margin status could play an important role.

Until now, we have focused our attention on a detailed presentation of the results related to our review on oncological outcomes and prognostic factors after CRC PALNMs resection. Although it does not represent the main purpose of our study, we think it is useful to complete our manuscript with the presentation of two interesting surgical endpoints, namely, indications adopted for PALND and description of postoperative complications.

According to the authors, indications for PALND stemmed from suspicious radiological findings [5,10,11,12,13,14,15,16,17]. In particular, we identified a computed tomography scan as the main performed imaging, followed by ^18^F-FDG positron emission tomography [5,10,11,12,13,14,15,16,17]. Computed tomography indicated that criteria for malignancy of lymph nodes included >5–8 mm short-axis, irregular margin and/or heterogenic contrast pattern/central necrosis (see Supplementary material-Table S3) [12,14,15,16]. However, scientific literature does not express consensus on radiological criteria to be adopted for PALND [15]. Moreover, in different cases, pathological PALNM positivity did not align with the radiological suspicion [5,10,11,12,13,14,15,16,17,29,30]. Therefore, several PALNDs could collect pathologically negative PALNs for malignancy [5,10,11,12,13,14,15,16,17,29,30]. For the above mentioned reasons, we only took into account patient populations with pathologically positive PALNMs, in order to recover long-term outcomes reducing the bias.

Furthermore, there is no consensus on the best anatomical area to be dissected [15]. Although all Authors define PALND as a surgical method for the recovery of PALNs-without going into further details, ruling out significant biases related to surgical approaches is almost impossible [5,10,11,12,13,14,15,16,17].

Finally, we tried to identify both the number and type of complications following PALND (see Supplementary material-Table S4). However, only five out of the nine included studies reported such information and recorded a 19–47% rate of complications, thus perfectly in line with previous reports in the scientific literature [5,10,11,13,17].

### Limitations

Our systematic review has some limitations, including: (i) the literature search did not include non-English-written scientific papers, except for studies in the Italian language; (ii) reported events were mainly part of small retrospective series; (ii) populations under analysis showed heterogeneity; (iii) the study time frame witnessed variation of diagnostic methods, surgical techniques and skills, perioperative CT/CRT protocols; (iv) many relevant data were not thoroughly described by the Authors, as reported in Table 1, Table 2, Table 3, Table 4 and Table 5; (v) many data were reported through median or mean results. All these reasons made direct comparison of findings arduous.

## 5. Conclusions

Currently, an agreement on the best treatment for patients with isolated PALNM from CRC has not been reached. Curative CRT alone may represent an option for poor surgical patients or those patients who show a poor performance. Preoperative CT or CRT followed by surgery should be taken into account in superselected patients who are likely to benefit from PALNM resection. However, although PALNMs resection in CRC patients may be considered as a feasible and beneficial option, no conclusions or recommendations can be made taking into account the current evidence.

Moreover, recurrence rates after PALNM resection seem to be high.

Therefore, further randomized, possibly multicenter trials are strongly recommended and mandatory if we want to confirm our results, and patient selection criteria need to be clearly identified.

## Figures and Tables

**Table 4:** Long-term outcomes and recurrences.

Author/Year	3-Year OS, %	5-Year OS, %	OS, Median Months (Range)	3-Year DFS, %	5-Year DFS, %	DFS, Median Months (Range)	Follow-Up, Median Months (Range)	Recurrence, *n* (%)	Sites, *n* (%)										
									Liver	Lung	Peritoneum	Distant LN	PALN	Bone	Brain	Local	Ovary	Mediastinum	Other
Min et al./2008	/	/	34	/	/	22	30	6 (100)	6 (100)	3 (50.0)	0	0	0	2 (33.3)	0	0	0	0	0
Choi et al./2010	59.4	53.4; 56.6 (CSS)	64 (17–111)	49	22	14	29 (7–75)	16 (66.7)	4 (16.7)	3 (12.5)	3 (12.5)	5 (20.8)	7 (29.2)	3 (12.5)	1 (4.2)	0	0	0	0
Gagnière et al./2015	68	56	/	61	51	/	85 (4–142)	/	/	/	/	/	/	/	/	/	/	/	/
Song et al./2016	65.7	/	30.2 (9.1–94.2)	40.2	/	20.3 (2.5–94.2)	31 (9.1–103.1)	9 (56.3)	3 (18.8)	2 (12.5)	2 (12.5)	2 (12.5)	4 (25.0)	1 (6.2)	0	2 (12.5)	0	0	0
Ogura et al./2017	/	70.3 (CSS)	/	/	60.5	/	58.8 (2.4–103.2)	7 (43.8)	2 (12.5)	0	1 (6.2)	2 (12.5)	4 (25.0)	0	0	0	0	0	0
Bae et al./2018	/	33.9	37 (6–169)	/	26.5	/	/	33 (67.3)	8 (16.3)	10 (20.4)	2 (4.1)	0	8 (16.3)	1 (2.0)	0	4 (8.2)	0	0	2 (4.1)
Yamamoto et al./2019	/	/	25 (2–44)	/	/	17 (2–44)	/	3 (60)	1 (20.0)	1 (20.0)	0	1 (20.0)	1 (20.0)	0	0	0	0	0	2 (40.0)
Kim et al./2020	/	87.5	84 (32–182)	/	0	24 (6–46)	50 (30–72)	8 (50)	0	2 (12.5)	0	5 (31.2)	0	0	0	1 (6.2)	0	0	0
Sakamoto et al./2020	63.2	/	/	26.3	/	/	30 (1.5–20.7)	/	/	/	/	/	/	/	/	/	/	/	/

OS = Overall survival; DFS = Disease-free survival; LN = Lymph node; PALN = Para-aortic lymph node; CSS = Cancer-specific survival.

**Table 5:** Prognostic factors reported by literature.

Author/Year	Univariate						Multivariate					
	OS	Overall Effect Size	95% CI of Overall Effect	DFS	Overall Effect Size	95% CI of Overall Effect	OS	Overall Effect Size	95% CI of Overall Effect	DFS	Overall Effect Size	95% CI of Overall Effect
Min et al./2008	Poorly/Mucinous primary tumor grade	/	/	/	/	/	Poorly/Mucinous primary tumor grade	HR = 2.844	1.037–7.797	/	/	/
	PALNM resection	/	/									
	Location i-PALNM	/	/									
Choi et al./2010	PALNM ≥ 3	/	/	PALNM ≥ 3	/	/	None	n/a	n/a	None	n/a	n/a
Gagnière et al./2015	Primary TNM Stage III	/	/	None	n/a	n/a	/	/	/	/	/	/
Song et al./2016	Primary TNM Stage IV	/	/	Primary TNM Stage IV	/	/	/	/	/	/	/	/
				PALNM ≥ 4	/	/						
Ogura et al./2017	None	n/a	n/a	Total LNM ≥ 7	/	/	/	/	/	/	/	/
				Regional LNM ≥ 4	/	/						
Bae et al./2018	PALNM > 7	/	/	PALNM > 7	/	/	PALNM > 7	HR = 3.291	1.309–8.275	PALNM > 7	HR = 2.484	0.993–6.211
	Preoperative CEA > 5	/	/	Preoperative CEA > 5	/	/				Preoperative CEA > 5	HR = 1.953	0.940–4.057
Yamamoto et al./2019	/	/	/	/	/	/	/	/	/	/	/	/
Kim et al./2020	DFI < 24 mo	HR = 0.457	0.218–0.958	/	/	/	DFI < 24 mo	HR = 0.321	0.125–0.821	/	/	/
	Primary pN	HR = 4.296	1.554–11.875	/	/	/	Primary pN	HR = 4.062	1.365–12.090	/	/	/
	PALNM resection	HR = 0.347	0.160–0.752	/	/	/	PALNM resection	HR = 0.379	0.151–0.955	/	/	/
Sakamoto et al./2020	Poorly/Mucinous primary tumor grade	HR = 4.21	1.67–10.6	pM1 b/c	HR = 3.59	1.15–11.21	Poorly/Mucinous primary tumor grade	HR = 7.18	2.21–23.4	pM1 b/c	HR = 2.49	1.05–5.90
	pM1 b/c	HR = 3.01	1.19–7.65				pM1 b/c	HR = 5.15	1.52–17.5			
	PALNM ≥ 4	HR = 2.81	1.07–7.39									

OS = Overall survival; DFS = Disease-free survival; PALNM = Para-aortic lymph node metastases; DFI = Disease-free interval; n/a = not applicable.

## Data Availability

The data presented in this study are available on request from the corresponding author.

## Supplementary Materials

The following supporting information can be downloaded at: https://www.mdpi.com/article/10.3390/cancers14030661/s1, Table S1: Newcastle-Ottawa Quality Assessment Form for Cohort Studies; Table S2: Chemotherapy regimens of included studies; Table S3: Indications adopted for PALND; Table S4: Postoperative complications.

## References

[1] Zizzo M., Galeone C., Braglia L., Ugoletti L., Siciliani A., Nachira D., Margaritora S., Pedrazzoli C., Paci M., Lococo F. (2020). Long-Term Outcomes after Surgical Resection for Synchronous or Metachronous Hepatic and Pulmonary Colorectal Cancer Metastases. Digestion.

[2] Wang X.Y., Zhang R., Wang Z., Geng Y., Lin J., Ma K., Zuo J.L., Lu L., Zhang J.B., Zhu W.W. (2019). Meta-analysis of the association between primary tumour location and prognosis after surgical resection of colorectal liver metastases. Br. J. Surg..

[3] Zarzavadjian Le Bian A., Genser L., Tabchouri N., Fillol C., Laforest A., Tresallet C., Ouaissi M., Fuks D. (2020). Abdominal lymph node recurrence from colorectal cancer: Resection should be considered as a curative treatment in patients with controlled disease. Surg. Oncol..

[4] Liberati A., Altman D.G., Tetzlaff J., Mulrow C., Gøtzsche P.C., Ioannidis J.P., Clarke M., Devereaux P.J., Kleijnen J., Moher D. (2009). The PRISMA statement for reporting systematic reviews and meta-analyses of studies that evaluate health care interventions: Explanation and elaboration. PLoS Med..

[5] Gagnière J., Dupré A., Chabaud S., Peyrat P., Meeus P., Rivoire M. (2015). Retroperitoneal nodal metastases from colorectal cancer: Curable metastases with radical retroperitoneal lymphadenectomy in selected patients. Eur. J. Surg. Oncol..

[6] Committee on Classification of Regional Lymph Nodes of Japan Society of Clinical Oncology (2003). Classification of regional lymph nodes in Japan. Int. J. Clin. Oncol..

[7] Sasaki K., Nozawa H., Kawai K., Hata K., Tanaka T., Nishikawa T., Shuno Y., Kaneko M., Murono K., Emoto S. (2020). Management of isolated para-aortic lymph node recurrence of colorectal cancer. Surg. Today.

[8] Wong J.S., Tan G.H., Teo M.C. (2016). Management of para-aortic lymph node metastasis in colorectal patients: A systemic review. Surg. Oncol..

[9] The Newcastle-Ottawa Scale (NOS) for Assessing the Quality of Nonrandomised Studies in Meta-Analyses. http://www.ohri.ca/programs/clinical_epidemiology/oxford.asp.

[10] Min B.S., Kim N.K., Sohn S.K., Cho C.H., Lee K.Y., Baik S.H. (2008). Isolated paraaortic lymph-node recurrence after the curative resection of colorectal carcinoma. J. Surg. Oncol..

[11] Choi P.W., Kim H.C., Kim A.Y., Jung S.H., Yu C.S., Kim J.C. (2010). Extensive lymphadenectomy in colorectal cancer with isolated para-aortic lymph node metastasis below the level of renal vessels. J. Surg. Oncol..

[12] Song S.H., Park S.Y., Park J.S., Kim H.J., Yang C.S., Choi G.S. (2016). Laparoscopic para-aortic lymph node dissection for patients with primary colorectal cancer and clinically suspected para-aortic lymph nodes. Ann. Surg. Treat Res..

[13] Ogura A., Akiyoshi T., Takatsu Y., Nagata J., Nagasaki T., Konishi T., Fujimoto Y., Nagayama S., Fukunaga Y., Ueno M. (2017). The significance of extended lymphadenectomy for colorectal cancer with isolated synchronous extraregional lymph node metastasis. Asian J. Surg..

[14] Bae S.U., Hur H., Min B.S., Baik S.H., Lee K.Y., Kim N.K. (2018). Which Patients with Isolated Para-aortic Lymph Node Metastasis Will Truly Benefit from Extended Lymph Node Dissection for Colon Cancer?. Cancer Res. Treat.

[15] Yamamoto S., Kanai T., Yo K., Hongo K., Takano K., Tsutsui M., Nakanishi R., Yoshikawa Y., Nakagawa M. (2019). Laparoscopic para-aortic lymphadenectomy for colorectal cancer with clinically suspected lymph node metastasis. Asian J. Endosc. Surg..

[16] Kim Y.I., Park I.J., Park J.H., Kim T.W., Ro J.S., Lim S.B., Yu C.S., Kim J.C. (2020). Management of isolated para-aortic lymph node recurrence after surgery for colorectal cancer. Ann. Surg. Treat Res..

[17] Sakamoto J., Ozawa H., Nakanishi H., Fujita S. (2020). Oncologic outcomes after resection of para-aortic lymph node metastasis in left-sided colon and rectal cancer. PLoS ONE.

[18] Jin M., Frankel W.L. (2018). Lymph Node Metastasis in Colorectal Cancer. Surg. Oncol. Clin. N. Am..

[19] Kim H.J., Choi G.S. (2019). Clinical Implications of Lymph Node Metastasis in Colorectal Cancer: Current Status and Future Perspectives. Ann. Coloproctol..

[20] Albandar M.H., Cho M.S., Bae S.U., Kim N.K. (2016). Surgical management of extra-regional lymph node metastasis in colorectal cancer. Exp. Rev. Anticancer Ther..

[21] Mengardo V., Bencivenga M., Weindelmayer J., Pavarana M., Giacopuzzi S., de Manzoni G. (2018). Para-aortic lymphadenectomy in surgery for gastric cancer: Current indications and future perspectives. Updates Surg..

[22] Dong Y.P., Deng J.Y. (2020). Advances in para-aortic nodal dissection in gastric cancer surgery: A review of research progress over the last decade. World J. Clin. Cases.

[23] Zizzo M., Castro Ruiz C., Annessi V., Zanelli M. (2020). Prognostic role of pancreatic head cancer metastatic paraaortic lymph nodes detected intraoperatively. HPB.

[24] Ho T.W., Mack L.A., Temple W.J. (2011). Operative salvage for retroperitoneal nodal recurrence in colorectal cancer: A systematic review. Ann. Surg. Oncol..

[25] Kim M.S., Cho C.K., Yang K.M., Lee D.H., Moon S.M., Shin Y.J. (2009). Stereotactic body radiotherapy for isolated paraaortic lymph node recurrence from colorectal cancer. World J. Gastroenterol..

[26] Yeo S.G., Kim D.Y., Kim T.H., Jung K.H., Hong Y.S., Kim S.Y., Park J.W., Choi H.S., Oh J.H. (2010). Curative chemoradiotherapy for isolated retroperitoneal lymph node recurrence of colorectal cancer. Radiother. Oncol..

[27] Lee J., Chang J.S., Shin S.J., Lim J.S., Keum K.C., Kim N.K., Ahn J.B., Kim T.I., Koom W.S. (2015). Incorporation of radiotherapy in the multidisciplinary treatment of isolated retroperitoneal lymph node recurrence from colorectal cancer. Ann. Surg. Oncol..

[28] Isozaki Y., Yamada S., Kawashiro S., Yasuda S., Okada N., Ebner D., Tsuji H., Kamada T., Matsubara H. (2017). Carbon-ion radiotherapy for isolated para-aortic lymph node recurrence from colorectal cancer. J. Surg. Oncol..

[29] Ogawa S., Itabashi M., Inoue Y., Ohki T., Bamba Y., Koshino K., Nakagawa R., Tani K., Aihara H., Kondo H. (2021). Lateral pelvic lymph nodes for rectal cancer: A review of diagnosis and management. World J. Gastrointest. Oncol..

[30] Zizzo M., Zanelli M., Sanguedolce F., Soriano A., Ascani S. (2021). Comment on “Is the Never-Ending Story Still Unsolved? Beyond the Long Debate About Lateral Pelvic Lymph Node Dissection in Rectal Cancer”. Dis. Colon Rectum..

