# Supplementary material for: Long-Term Outcomes of Surgical Resection of Pathologically Confirmed Isolated Para-Aortic Lymph Node Metastases in Colorectal Cancer: A Systematic Review
Source: Cancers (Basel). 2022 Jan 28;14(3):661. doi: 10.3390/cancers14030661 (PMC8833834; doi:10.3390/cancers14030661)
Supplement: Supplementary file 1 [file cancers-14-00661-s001.zip › cancers-1538539-supplementary.pdf]

cancers

MDPI

Long-Term Outcomes of Surgical Resection of Pathologically Confirmed Isolated Para-Aortic Lymph Node Metastases in Colorectal Cancer: A Systematic Review

Maurizio Zizzo, Maria Pia Federica Dorma, Magda Zanelli, Francesca Sanguedolce, Maria Chiara Bassi, Andrea Palicelli, Stefano Ascani and Alessandro Giunta

Table S1. Newcastle-Ottawa Quality Assessment Form for Cohort Studies.

Author/Year	Newcastle-Ottawa Scale									Total	Quality
	Selection				Comparability		Outcome				
	1[a]	2[b]	3[c]	4[d]	5[e]	6[f]	7[g]	8[h]	9[i]		
Min et al/2008 [10]	●	●	●	●	●	●	●	●	●	9	Good
Choi et al/2010 [11]	●	●	●	●	●	●	●	●	●	9	Good
Gagnière et al/2015 [5]	●	●	●	●	●	●	●	●	●	9	Good
Song et al/2016 [12]	●	●	●	●	●	●	●	●	●	9	Good
Ogura et al/2017 [13]	●	●	●	●	●	●	●	●	●	9	Good
Bae et al/2018 [14]	●	●	●	●	●	●	●	-	-	7	Poor
Yamamoto et al/2019 [15]	●	●	●	●	●	●	●	-	-	7	Poor
Kim et al/2020 [16]	●	●	●	●	●	●	●	●	●	9	Good
Sakamoto et al/2020 [17]	●	●	●	●	●	●	●	●	●	9	Good

[a] Representativeness of the exposed cohort; [b] Selection of the non-exposed cohort; [c] Ascertainment of exposure; [d] Demonstration that outcome of interest was not present at start of study; [e] Comparability of cohorts on the basis of the design or analysis (adjusted for age); [f] Comparability of cohorts on the basis of the design or analysis (adjusted for any other factor); [g] Assessment of outcome; [h] Was follow-up long enough for outcomes to occur; [i] Adequacy of follow-up of cohorts.

Table S2. Chemotherapy regimens of included studies.

**Author/Year**	**Chemotherapy, n**			**Chemotherapy regimen, n**										
	**No**	**Neoadjuvant**	**Adjuvant**	**Neoadjuvant**		**Adjuvant**								
				**Not reported**	**5-Fluorouracil/Oxaliplatin+Target agent**	**Not reported**	**Doxifluridine**	**CPT-11 based**	**Oxaliplatin**	**5-Fluorouracil/Leucovorin/Oxaliplatin**	**Capecitabine**	**5-Fluorouracil/Leucovorin**	**5-Fluorouracil/Oxaliplatin**	**Capecitabine/Oxaliplatin**
Min et al/2008	0	0	6	n/a	n/a	0	0	0	0	6	0	0	0	0
Choi et al/2010	1	0	23	n/a	n/a	0	0	10		0	13		/	/
Gagnière et al/2015	0	/	/	/	/	/	/	/	/	/	/	/	/	/
Song et al/2016	0	0	16	n/a	n/a	1	1	/	/	0	1	4	3	6
Ogura et al/2017	0	4	15	3	1	5	/	10		/	0	0	/	/
Bae et al/2018	/	/	47	/	/	0	0	0	0	0	0	47		0
Yamamoto et al/2019	0	0	5	n/a	n/a	5	/	/	/	/	/	/	/	/
Kim et al/2020	3	0	13	n/a	n/a	13	/	/	/	/	/	/	/	/
Sakamoto et al/2020	10	0	9	n/a	n/a	9	/	/	/	/	/	/	/	/

[n] Number; [n/a] Not applicable.

Table S3. Indications adopted for PALND.

Author/Year	Patient population, n	CT			MRI			PET			Biopsy	
		**Yes/No**	**n**	**PALNM CT criteria**	**Yes/No**	**n**	**PALNM MRI criteria**	**Yes/No**	**n**	**PALNM PET criteria**	**Yes/No**	**n**
Min et al/2008	6	Yes	/	/	Yes	/	/	Yes	/	High 18-FDG uptake	Yes	/
Choi et al/2010	24	Yes	24	/	Yes	/	/	Yes	/	High 18-FDG uptake	Yes	/
Gagnière et al/2015	10	Yes	/	/	No	0	n/a	Yes	/	High 18-FDG uptake	No	0
Song et al/2016	16	Yes		short-axis diameter > 8 mm<br>irregular margin<br>central necrosis	No	0	n/a	Yes	/	High 18-FDG uptake	No	0
Ogura et al/2017	16	Yes	16	/	No	0	n/a	Yes	/	High 18-FDG uptake	No	0
Bae et al/2018	49	Yes	/	short-axis diameter > 5 mm<br>irregular margin<br>heterogenic contrast pattern	No	0	n/a	Yes	/	High 18-FDG uptake	No	0
Yamamoto et al/2019	5	Yes	5	short-axis diameter > 8 mm<br>irregular margin<br>heterogenic contrast pattern	No	0	n/a	No	0	n/a	No	0
Kim et al/2020	16	Yes	/	short-axis diameter > 8 mm	Yes	/	short-axis diameter > 8 mm	Yes	/	Visible LN < 8 mm<br>High 18-FDG uptake	No	0
Sakamoto et al/2020	19	Yes	/	/	No	0	n/a	Yes	/	High 18-FDG uptake	No	0

n Number; CT Computed tomography; MRI Magnetic resonance imaging; PET Positron emission tomography; PALNM Para-aortic lymph node metastasis; mm millimiter; FDG Fluorodeoxyglucose; n/a Not applicable.

Table S4. Postoperative complications.

Author/Year	Patient population, n	Complications, n (%)	Clavien-Dindo, n					Types of Complications (n)
			**Grade I**	**Grade II**	**Grade III**	**Grade IV**	**Grade V**	
Min et al/2008	6	2 (33)	/	/	/	/	/	Intestinal obstruction (2)
Choi et al/2010	24	5 (21)	/	/	/	/	/	Wound infection (3), Ileus (1), Bleeding (1)
Gagnière et al/2015	10	2 (20)	0	0	2		0	Pulmonary embolism (1), Right ureter injury (1)
Song et al/2016	16	/	/	/	/	/	/	/
Ogura et al/2017	16	3 (19)	/	/	/	/	/	Ileus (1), Bleeding (1), Pelvic dead space infection (1)
Bae et al/2018	49	/	/	/	/	/	/	/
Yamamoto et al/2019	5	/	/	/	/	/	/	/
Kim et al/2020	16	/	/	/	/	/	/	/
Sakamoto et al/2020	19	9 (47)	1	5	3	0	0	Urinary retention (1), SSI (3), Intra-abdominal abscess (1), Atelectasis (1), Delayed gastric emptying (1), Paralytic ileus (1), Anastomotic leakage (1)

^{n} Number.
